# Association between health examination items and body mass index among school children in Hualien, Taiwan

**DOI:** 10.1186/1471-2458-13-975

**Published:** 2013-10-19

**Authors:** Chia-Hsiang Chu, Jen-Hung Wang, Rong-Hwa Jan, Chih-Hao Huang, Ching-Feng Cheng

**Affiliations:** 1 Department of Pediatrics, Tzu Chi General Hospital and Tzu Chi University, Hualien, Taiwan; 2 Department of Medical Research, Tzu Chi General Hospital, Hualien, Taiwan; 3 Institute of Biomedical Sciences, Academia Sinica, Taipei, Taiwan; 4 Department of Dentistry, Tzu Chi General Hospital, Hualien, Taiwan

**Keywords:** Body mass index, School children, Health examination

## Abstract

**Background:**

To assess the prevalence of obesity and major physical examination items including dental caries, myopia, pinworm, hematuria, and proteinuria among school children in Hualien, Taiwan. In addition, the health status differences between gender, grader, levels of residence urbanization, and body mass index (BMI) were examined.

**Methods:**

Cross-sectional studies with a total of 11,080 students (age, 7–14 years) in grades 1, 4, and 7 were evaluated for weight, height, routine physical examination, and urine analysis during the 2010 Student Health Examination in Hualien. Frequencies, Chi-square test, and logistic regression were conducted using SPSS.

**Results:**

Of the 11,080 students evaluated, 1357 (12.2%) were overweight, and 1421 (12.8%) were obese. There were significant differences in overweight/obese prevalence by gender, by grader, and by levels of residence urbanization. Dental caries, myopia, and obesity were the most prevalent health problems among these students (75.6%, 33.0%, and 12.8%, respectively). In crude and adjusted analyses, research results showed that there were significant differences in the prevalence of major physical examination items between different gender, grader, levels of residence urbanization, and BMI groups. Girls had a higher prevalence of dental caries, myopia, and hematuria than boys (all p < 0.01), whereas boys had a higher prevalence of pinworm than girls (p = 0.02). Students in higher grades had significantly higher prevalence of myopia, hematuria, and proteinuria (all p < 0.01), whereas students in lower grades had higher prevalence of dental caries and pinworm (p < 0.01). Students with abnormal BMI had lower prevalence of pinworm (p < 0.01). Students residing in suburban and rural areas had higher prevalence of dental caries, pinworm, and hematuria (all p < 0.01), and lower prevalence of myopia than students residing in urban areas (all p < 0.01).

**Conclusion:**

Routine health examination provides an important way to detect students’ health problems. Our study elucidated major health problems among school children in Hualien, Taiwan. In addition, the results also indicated that the prevalence of health problems had a significant relationship with gender, grader, levels of residence urbanization, and BMI. It is suggested that school health interventions should consider students’ health profiles along with their risk factors status in planning.

## Background

An increasing prevalence of obesity in both adults and children has been observed in many countries throughout the world, including Taiwan [1,2]. Studies show that in addition to the growing problem of excess weight, there is an escalation of related complications as overweight children reach adulthood [3]. The strong association between childhood obesity and adult diseases brings to light the importance of early detection and suggests that prevention and treatment of childhood obesity should be pursued to reduce morbidity and mortality in adulthood [4-6]. In addition, good health is vital to a child’s ability to learn and succeed in life because diseases or health problems can prevent a child from completely engaging in learning activities [7].

As a means of primary prevention, many studies suggest that physical examinations provide an avenue for identifying high-risk groups among school-aged children and also provide clues for secondary prevention [8,9]. Thus, some schools require physical examination before enrollment or sport activities [10]. Physical examinations initiated when children are of elementary school age can provide a good means for tracking physical development and health problems, which can in turn provide parents, schools, and health professionals more information for use in the early prevention and treatment of these problems [11]. One study has shown that dental caries, myopia, and obesity are the most prevalent health problems among 1st graders of public elementary schools [12]. In addition, the prevalence of pinworms, which is the most common human intestinal parasite, is relatively high in Hualien (5.79% and 6.25% in 2007 and 2008, respectively) compared with other cities in Taiwan (range, 1.53-2.23%) [13]. Detection of hematuria and proteinuria can indicate renal or bladder disease [14].

The objective of this study was to assess the prevalence of obesity and major physical examination items including dental caries, myopia, pinworm, hematuria, and proteinuria among school children in Hualien, Taiwan. In addition, the health status differences between gender, grader, levels of residence urbanization, and body mass index (BMI) were examined.

## Methods

### Subjects

The data used in this comparative descriptive study were collected in the fall of 2010 from 1st (aged 7–8 years) and 4th (aged 10–11 years) graders of all elementary schools and 7th graders (aged 13–14 years) of all junior high schools in Hualien County, Taiwan. A total of 11,080 students from 106 elementary schools and 25 junior high schools were included in the study. The completion rate was 99.1% (11,080 out of 11,179). The study was reviewed and approved by the Hualien County Government Education Bureau. An approval certificate for this study was also issued from our Research Ethics Committee (REC No.: IRB101-125). Informed written consent was obtained from the students’ parents or guardians. The parents or guardians were required to accompany their children to the physical examinations, and the students were notified to wear suitable clothing. The consent form, offered by the Hualien County Government Education Bureau, informed parents/guardians that these examinations were non-intrusive with minimum risk and students or parent/guardians were free to withdraw from any examination item at any time.

### Measurements & procedures

A physical examination team from Tzu Chi General Hospital including pediatricians, dentists, oculists, and nurses was employed to conduct physical examinations in the health center of each school. Areas assessed included eyes, teeth, ears-nose-throat (ENT), heart-lung-abdomen, bones-muscle, reproductive-urinary, skin and other systems grouped by the regulations set by the Taiwan Ministry of Education [5]. This study assessed the prevalence of obesity and major physical examination items including dental caries, myopia, pinworm, hematuria, and proteinuria among school children in Hualien, Taiwan. In addition, the health status differences between gender, grader, levels of residence urbanization, and body mass index (BMI) were examined. Population density (person per square kilometer, km^2^) is the major indicator for the urbanization level. Residence urbanization was categorized into three groups, urban (above five hundred), suburban (ranged fifteen to below five hundred), and rural (below fifteen) according to population densities of residential areas [15]. Based on this categorization, the distribution of cities was 3(23%) urban, 7(54%) suburban, and 3(23%) rural. The conditions of being overweight and obese were defined on the basis of age-gender-specific BMI cut-points from Taiwanese national reference values for children and adolescents developed by the Department of Health (DOH) in Taiwan. Children were classified as underweight, normal, overweight, or obese. Myopia was defined as having spherical equivalents of ≦ − 0.8 D. A student was identified as having dental problem if he or she had ≧1 untreated cavity. Pinworm infection was confirmed if pinworm eggs were found on pinworm patch using an optical microscope. Hematuria, or blood in the urine, could be identified either grossly (visible) or microscopically (as defined by >3-5 red blood cells per high power field when viewed under magnification). Proteinuria was defined as presence of ≧100 mg/dL protein in early morning urine.

### Data analysis

Descriptive statistics for each physical examination items such as dental caries, myopia, pinworms, hematuria and proteinuria were presented as frequencies or proportions. A Chi-square test was performed to identify examination items that were significantly related to the BMI groups. Logistic regression models were used to simultaneously analyze the association between prevalence of physical examination items and risk factors encountered. Crude and adjusted odds ratios and 95% confidence intervals were calculated. Statistical significance was defined as a p value of < 0.05. All statistical analyses were performed using the statistical software SPSS, version 17.0 (SPSS Inc., Chicago, IL, USA).

## Results

This study explored the morbidity of common health problems among school-age children in Hualien and examined the health status differences between gender, grader, levels of residence urbanization, and body mass index (BMI). Of the 11,080 students (age, 7–14 years), 52.4% were boys (5,806) and 47.6% were girls (5,274). Overweight and obesity prevalence were 12.2% and 12.8% respectively. Table 1 indicated that the level of BMI had a significant association with gender, grader, and levels of urbanization (all p < 0.01). The prevalence rates of overweight and obese in boys were significantly higher than in girls. The prevalence rate of obesity increased with higher grades. The prevalence rates of normal BMI in the urban and suburban areas (56.3% and 57.2% respectively) were significantly lower than in the rural area (65.4%). Table 2 contained the morbidity of common health problems and their association with BMI. Dental caries, myopia, hematuria, pinworm, proteinuria were the most prevalent health problems (75.6%, 33.0%, 3.8%, 3.1%, and 2.4%, respectively). To clarify whether BMI was associated with a higher risk of abnormal for common health problems, logistic regression with consideration of the factors, including gender, grader, levels of residence urbanization, and body mass index (BMI), simultaneously was performed. Analysis results were summarized in Table 3 and described individually for each condition.

**Table 1:** Demographic information among students accepting physical examination

**Variable**	**N**	**Underweight(%)**	**Normal(%)**	**Overweight(%)**	**Obese(%)**	**P-value**
Overall	11080	1973(17.8)	6329(57.1)	1357(12.2)	1421(12.8)	NA
Gender						<0.01*
Boy	5806	1011(17.4)	3243(55.9)	735(12.7)	817(14.1)	
Girl	5274	962(18.2)	3086(58.5)	622(11.8)	604(11.5)	
Grader						<0.01*
1	3060	597(19.5)	1910(62.4)	292(9.5)	261(8.5)	
4	3794	727(19.2)	2065(54.4)	523(13.8)	479(12.6)	
7	4226	649(15.4)	2354(55.7)	542(12.8)	681(16.1)	
Urbanization						<0.01*
Urban	7596	1416(18.6)	4280(56.3)	950(12.5)	950(12.5)	
Suburban	2805	486(17.3)	1605(57.2)	320(11.4)	394(14.0)	
Rural	679	71(10.5)	444(65.4)	87(12.8)	77(11.3)	

*P-value < 0.05 was considered statistically significant after Chi-square test.

**Table 2:** Prevalence rates of most common health problems and their association with BMI

**Variable**	**No.(%)**	**Underweight(%)**	**Normal(%)**	**Overweight(%)**	**Obese(%)**	**P-value**
Caries						<0.01*
Abnormal	8373(75.6)	1522(77.1)	4816(76.1)	1007(74.2)	1028(72.3)	
Normal	2707(24.4)	451(22.9)	1513(23.9)	350(25.8)	393(27.7)	
Myopia^a^						0.03*
Abnormal	3532(33.0)	629(32.8)	1953(32.1)	467(35.7)	483(35.0)	
Normal	7164(67.0)	1286(67.2)	4140(67.9)	840(64.3)	898(65.0)	
Pinworm^b^						<0.01*
Abnormal	339(3.1)	80(4.1)	215(3.5)	24(1.8)	20(1.5)	
Normal	10447(96.9)	1850(95.9)	5952(96.5)	1300(98.2)	1345(98.5)	
Hematuria^c^						0.08
Abnormal	413(3.8)	60(3.1)	230(3.7)	63(4.7)	60(4.3)	
Normal	10494(96.2)	1886(96.9)	5992(96.3)	1278(95.3)	1338(95.7)	
Proteinuria^c^						0.05
Abnormal	260(2.4)	43(2.2)	165(2.7)	32(2.4)	20(1.4)	
Normal	10647(97.6)	1903(97.8)	6057(97.3)	1309(97.6)	1378(98.6)	

*P-value < 0.05 was considered statistically significant after Chi-square test. a: n = 10696, b: n = 10786, c: n = 10907.

**Table 3:** Factors associated with a higher risk of abnormal for major screening items

	**Caries**			**Myopia**			**Pinworm**		
	**Crude**	**Adjusted**^**a**^		**Crude**	**Adjusted**^**a**^		**Crude**	**Adjusted**^**a**^	
**Variable**	**OR(95% CI)**	**OR(95% CI)**	**P-value**	**OR(95% CI)**	**OR(95% CI)**	**P-value**	**OR(95% CI)**	**OR(95% CI)**	**P-value**
Gender									
Boy	1	1		1	1		1	1	
Girl	1.14(1.05, 1.25)	1.14(1.04, 1.24)	<0.01*	1.29(1.19, 1.40)	1.37(1.25, 1.49)	<0.01*	0.77(0.62, 0.97)	0.76(0.61, 0.95)	0.02*
Grader									
1	1	1		1	1		1	1	
4	0.61(0.54, 0.69)	0.61(0.54, 0.69)	<0.01*	3.59(3.17, 4.07)	3.68(3.24, 4.18)	<0.01*	0.56(0.45, 0.70)	0.58(0.47, 0.73)	<0.01*
7	0.40(0.36, 0.45)	0.41(0.36, 0.46)	<0.01*	5.91(5.22, 6.68)	6.00(5.29, 6.81)	<0.01*	NA	NA	NA
BMI									
Underweight	1	1		1	1		1	1	
Normal	0.94(0.84, 1.06)	0.95(0.84, 1.08)	0.43	0.96(0.86, 1.08)	0.98(0.87, 1.10)	0.75	0.89(0.68, 1.16)	0.78(0.60, 1.02)	0.07
Overweight	0.85(0.73, 1.00)	0.91(0.77, 1.07)	0.27	1.14(0.98, 1.32)	1.04(0.89, 1.22)	0.62	0.47(0.30, 0.75)	0.45(0.28, 0.71)	<0.01*
Obese	0.78(0.66, 0.91)	0.86(0.73, 1.01)	0.07	1.10(0.95, 1.27)	0.96(0.82, 1.12)	0.63	0.44(0.26, 0.72)	0.42(0.26, 0.70)	<0.01*
Urbanization									
Urban	1	1		1	1		1	1	
Suburban	1.34(1.21, 1.49)	1.34(1.21, 1.49)	<0.01*	0.47(0.43, 0.52)	0.44(0.39, 0.49)	<0.01*	1.93(1.50, 2.49)	1.94(1.50, 2.50)	<0.01*
Rural	1.40(1.16, 1.70)	1.19(0.98, 1.45)	0.08	0.32(0.26, 0.39)	0.39(0.31, 0.48)	<0.01*	4.76(3.58, 6.33)	4.85(3.63, 6.48)	<0.01*
Hematuria				Proteinuria					
Gender									
Boy	1	1		1	1				
Girl	2.25(1.83, 2.77)	2.26(1.84, 2.79)	<0.01*	0.91(0.72, 1.17)	1.07(0.84, 1.37)	0.59			
Grader									
1	1	1		1	1				
4	1.09(0.80, 1.48)	1.09(0.80, 1.49)	0.59	2.15(1.32, 3.53)	2.22(1.35, 3.63)	<0.01*			
7	2.67(2.04, 3.49)	2.92(2.22, 3.85)	<0.01*	6.27(4.02, 9.79)	6.50(4.15, 10.16)	<0.01*			
BMI									
Underweight	1	1		1	1				
Normal	1.21(0.90, 1.61)	1.09(0.81, 1.46)	0.56	1.21(0.86, 1.69)	1.15(0.82, 1.62)	0.42			
Overweight	1.55(1.08, 2.22)	1.41(0.98, 2.04)	0.06	1.08(0.68, 1.72)	0.97(0.61, 1.54)	0.89			
Obese	1.41(0.98, 2.03)	1.21(0.83, 1.75)	0.32	0.64(0.38, 1.10)	0.52(0.30, 0.89)	0.02*			
Urbanization									
Urban	1	1		1	1				
Suburban	1.69(1.36, 2.10)	1.70(1.37, 2.12)	<0.01*	0.92(0.69, 1.22)	0.94(0.71, 1.26)	0.70			
Rural	2.21(1.59, 3.09)	2.94(2.08, 4.15)	<0.01*	0.53(0.27, 1.04)	0.79(0.40, 1.56)	0.50			

^a^Adjusted for gender, grader, BMI, and urbanization. OR = odds ratio; CI = confidence interval. *P-value < 0.05 was considered statistically significant after logistic regression.

### Dental caries

Dental caries were the most serious problem with an overall prevalence of 75.6%. Gender, grader, and urbanization were found associated with dental caries. Girls had a significantly higher odds ratio (1.14) of dental caries compared with boys (p < 0.01). Students in higher grades (4th and 7th grades) had significantly lower odds ratios (0.61 and 0.41, respectively) of dental caries compared with students in 1st grade (all p < 0.01). Students in suburban and rural areas had significantly higher odds ratios (1.34 and 1.19, respectively) of dental caries compared with students in urban area.

### Myopia

The overall prevalence of myopia was 33.0%. Gender, grader, and urbanization were found associated with myopia. Girls had a significantly higher odds ratio (1.37) of myopia compared with boys (p < 0.01). Students in higher grades(4th and 7th grades) had significantly higher odds ratios (3.68 and 6.00, respectively) of myopia compared with students in 1st grade (all p < 0.01). Students in suburban and rural areas had significantly lower odds ratios (0.44 and 0.39, respectively) of myopia compared with students in urban area (all p < 0.01).

### Pinworm

The overall prevalence of pinworm was 3.1%. Gender, grader, BMI, and urbanization were found associated with pinworm. Girls had a significantly lower odds ratio (0.76) of pinworm compared with boys (p = 0.02). Students in higher grade (4th grade) had significantly lower odds ratios (0.58) of pinworm compared with students in 1st grade (p < 0.01). Overweight and obese students had significantly lower odds ratios (0.45 and 0.42, respectively) of pinworm compared with underweight students (all p < 0.01). Students in suburban and rural areas had significantly higher odds ratios (1.94 and 4.85, respectively) of pinworm compared with students in urban area (all p < 0.01).

### Hematuria

The overall prevalence of hematuria was 3.8%. Gender, grader, and urbanization were found associated with hematuria. Girls had a significantly higher odds ratio (2.26) of hematuria compared with boys (p < 0.01). Students in 7th grade had a significantly higher odds ratio (2.92) of hematuria compared with students in 1st grade (p < 0.01). Students in suburban and rural areas had significantly higher odds ratios (1.70 and 2.94, respectively) of hematuria compared with students in urban area (all p < 0.01).

### Proteinuria

The overall prevalence of proteinuria was 2.4%. Grader and BMI were found associated with proteinuria. Students in higher grades (4th and 7th grades) had significantly higher odds ratios (2.22 and 6.50, respectively) of proteinuria compared with students in 1st grade (all p < 0.01). Obese students had a significantly lower odds ratio (0.52) of proteinuria compared with underweight students (p = 0.02).

## Discussion

Our 2010 school children health examination data from 1st, 4th, and 7th graders in Hualien County demonstrated that dental caries, myopia, and obesity were the top three health problems with prevalence rates of 75.6%, 33.0%, and 12.8%, respectively. These top three health problems are consistent with those reported by prior studies of elementary school children entrance health checks in Taipei County where the prevalence rates of dental caries, myopia, and obesity were 69.6%, 27.1%, and 9.5%, respectively [12]. These health problems associated with school children warrants further attention since the results of our study, which was conducted on a larger scale and covered students of different ages, indicate higher prevalence.

Our data also elucidate the relationship between prevalence of major health examination items and some risk factors, which were not mentioned in prior studies in Taiwan. We found that there were significant differences in the prevalence of major physical examination items between different gender, grader, levels of residence urbanization, and BMI groups. Girls had a higher prevalence of dental caries, myopia, and hematuria than boys, whereas boys had a higher prevalence of pinworm than girls. The gender differences of these health problems are also consistent (myopia: girls > boys; obesity: boys > girls) with prior studies except for the fact that girls had a higher prevalence of dental caries in Hualien [12,13]. It may be related to the habit of eating sweets and derivatives more commonly observed in girls than in boys [16].

Students in higher grades had significantly higher prevalence of myopia, hematuria, and proteinuria, whereas students in lower grades had higher prevalence of dental caries and pinworm. The correlation between students of higher grades with a higher prevalence of myopia can be explained by the heavy homework assignments for primary and secondary school students. The higher prevalence of hematuria in female students of higher grades may correlate with the possibility of contamination by menstrual blood. The higher prevalence of proteinuria in students of higher grades may correlate with long-term use of plastic containers for food and beverage. It increases the risk of exposure to bisphenol A [17]. The highest prevalence of dental caries was observed in 1st graders (84.4%), which correlates with the teeth changing period. It also reflects the common ideas that dental health in toddlers and young children does not receive much attention from traditional Taiwanese parents. The negative correlation between dental caries and age may also be attributed to the execution of proper teeth brushing after lunch at school.

Students with abnormal BMI had lower prevalence of pinworm. The pinworms are parasites and feed off of the food in the intestines. If a large number of pinworms are living in the intestine, they may ingest most of the food and nutrients the affected individual eats. This loss of nutrients may cause a person with pinworms to lose weight from the lack of nutrition.

Students residing in suburban and rural areas had higher prevalence of dental caries, pinworm, and hematuria, and lower prevalence of myopia than students residing in urban areas. Low level of urbanization often accompanies with low quantity of medical resource and poor sanitary condition. It may correlate with higher prevalence of dental caries, pinworm, and hematuria. One probable reason maybe the use of un-sanitized drinking water with increased calcium levels in the less urban areas, in which may increase the risk for hypercalciuria induced micro-hematuria seen in those children [18].

Several limitations and suggestions related to this study should be mentioned. First, our data identified health problems found in school children; however the severity of the diseases was not recorded. Nevertheless, referral notice was sent to parents so that the children could be followed-up in either medical centers or local clinics. Second, the year 2010 school children health examination data was collected for cross-sectional analysis, and thus, the directionality of causation cannot be established. However, our proper analysis of this cross-sectional data represents an useful initial step to identify associations between obesity and other major health problems. Third, our national policy includes only physical examination of 1st, 4th, and 7th graders; therefore, follow-up of these children 3 years later would be valuable to investigate the association of a continuous obese status with these health problems. Fourth, although we found a higher prevalence of hematuria among teenage girls in less urbanized areas, the exact etiology for these results is not clear and may require further follow-up in adolescent. Fifth, some studies have shown that parental socioeconomic status (SES) is correlated with children’s health, particularly worms and caries. However, it cannot be taken into account since parents’ information has not been investigated with consideration of privacy. SES related factors such as wealth and education statuses of children’s family are suggested to be included to explain the origins of the health problems in further studies. Finally, based on our findings, some health care interventions can be executed now with future evaluation of their effects.

## Conclusion

Routine health examination provides an important way to detect students’ health problems. Our study elucidated major health problems among school children in Hualien, Taiwan. Dental caries, myopia, and obesity are still the top three health problems in the school children of Hualien, Taiwan. It is worth noticing that the prevalence rates of major health problems was higher than those in prior studies. In addition, the results also indicated that the prevalence of health problems had a significant relationship with gender, grader, levels of residence urbanization, and BMI. It is suggested that school health interventions should consider students’ health profiles along with their risk factors status in planning.

## Competing interests

The authors declare that they have no competing interests.

## Authors’ contributions

CH Chu and CF Cheng had full access to all the data in the study and take responsibility for the integrity of the data and the accuracy of the data analysis. Study concept and design: CH Chu and CF Cheng. Acquisition of data: JH Wang, RH Jan, and CF Cheng. Analysis and interpretation of data: JH Wang, CH Huang, and CF Cheng. Manuscript writing: JH Wang and CF Cheng. All authors read and approved the final manuscript.

## Pre-publication history

The pre-publication history for this paper can be accessed here:

http://www.biomedcentral.com/1471-2458/13/975/prepub
